# Fullerenes Influence the Toxicity of Organic Micro-Contaminants to River Biofilms

**DOI:** 10.3389/fmicb.2018.01426

**Published:** 2018-07-03

**Authors:** Anna Freixa, Vicenç Acuña, Marina Gutierrez, Josep Sanchís, Lúcia H. M. L. M. Santos, Sara Rodriguez-Mozaz, Marinella Farré, Damià Barceló, Sergi Sabater

**Affiliations:** ^1^Catalan Institute for Water Research, Girona, Spain; ^2^Water and Soil Quality Research Group, Institute of Environmental Assessment and Water Research, Spanish National Research Council, Barcelona, Spain; ^3^Research Group on Ecology of Inland Waters, Institute of Aquatic Ecology, University of Girona, Girona, Spain

**Keywords:** carbon nanoparticles, pollutants, microbial ecotoxicology, mixtures, periphyton, diuron, triclosan, venlafaxine

## Abstract

Organic micro-contaminants (OMCs) enter in freshwaters and interact with other contaminants such as carbon nanoparticles, becoming a problem of unknown consequences for river ecosystems. Carbon nanoparticles (as fullerenes C_60_) are good adsorbents of organic contaminants and their interaction can potentially affect their toxicity to river biofilms. We tested the C_60_ interactions with selected OMCs and their effects on river biofilms in different short-term experiments. In these, river biofilms were exposed to C_60_ and three OMCs (triclosan, diuron, or venlafaxine) and their respective mixtures with fullerenes (C_60_ + each OMC). The effects were evaluated on structural, molecular, and functional descriptors of river biofilms. Our results showed that C_60_ did not cause toxic effects in river biofilms, whereas diuron and triclosan significantly affected the heterotrophic and phototrophic components of biofilms and venlafaxine affected only the phototrophic component. The joint exposure of C_60_ with venlafaxine was not producing differences with respect to the former response of the toxicant, but the overall response was antagonistic (i.e., decreased toxicity) with diuron, and synergistic (i.e., increased toxicity) with triclosan. We suggest that differences in the toxic responses could be related to the respective molecular structure of each OMC, to the concentration proportion between OMC and C_60,_ and to the possible competition between C_60_ pollutants on blocking the receptors of the biological cell membranes. We conclude that the presence of C_60_ at low concentrations modified the toxicity of OMC to river biofilms. These interactions should therefore be considered when predicting toxicity of OMC in river ecosystems.

## Introduction

Organic micro-contaminants (OMCs) and carbon nanoparticles enter in freshwater ecosystems *via* point (e.g., sewage discharge) and diffuse sources (e.g., run-off events) as well as from atmospheric depositions. The widespread use of carbon nanomaterials, in particular fullerenes (such as C_60_), has prompted the arrival of these nanomaterials to rivers. Concentrations of up to ng L^−1^ have been observed in effluents of wastewater treatment plants (Farré et al., 2010; Wang et al., 2010). C_60_ are molecules with 60 atoms of carbon forming fused hexagons and pentagons, and their unique properties (i.e., proportionately very large surface area) led to several uses in nanotechnology industry such as water treatment, medical applications, microelectronics, photovoltaic devices, and cosmetics (Bakry et al., 2007; Benn et al., 2011; Farré et al., 2011). When reaching freshwater systems, these nanomaterials may undergo transformations such as oxidation, or photo- and biological degradation. In addition, they can easily aggregate and participate in sorption processes with OMCs, organic matter, and aquatic organisms (Bundschuh et al., 2016).

Although environmental concentrations of C_60_ do not pose a direct threat on aquatic organisms, the co-occurrence of these materials with OMC can potentially modify their original availability (i.e., the degree of accessibility of every compound to the organisms) and their toxicity to river organisms (Freixa et al., 2018). The toxicity of OMC to them has been widely analyzed (Kuzmanović, 2015), and it is our assumption that C_60_ can interact with OMC both as carriers and enhancers of the toxicity of contaminants and as blinding their action and reducing their toxic effect. This variety of responses may produce additive, synergistic, or antagonistic interactions (Folt et al., 1999; Crain et al., 2008; Côté, et al., 2016). Some previous studies have reported either synergistic or antagonistic effects to bacteria, daphnids, or fish (Yang et al., 2010; Ferreira et al., 2014; Hu et al., 2015; Sanchís et al., 2016). Specifically, Baun et al. (2008) showed that the toxicity may vary depending on the toxicant, and observed that the toxicity to phenanthrene in the planktonic alga *Pseudokirchneriella subcapitata* increased in the presence of C_60_, but that of pentachlorophenol decreased. However, the patterns of toxicity responses to biofilm communities produced by conjoint C_60_ and OMC are still unclear and deserve detailed analysis.

Biofilms are complex communities of algae, bacteria, and fungi, all embedded within a polysaccharide matrix which contributes to the stability and protection of microorganisms (Gerbersdorf et al., 2008; Flemming and Wingender, 2010). Biofilms dominate the river microbial life and are particularly relevant as nutrient and organic matter recyclers (Battin et al., 2016). Biofilms as well are the early receivers and responders to the presence of OMC, mainly because of their position as interfaces between water and the sediments (Sabater et al., 2007). Most previous studies on the ecotoxicity of carbon nanomaterials (Freixa et al., 2018) mainly derive from single-species analyses, and only a few (e.g., Lawrence et al., 2016) approach the response of such a complex consortium of microorganisms as those constituted by biofilms.

In this paper, we aim to ascertain the interactive effects of fullerenes on the toxicity of selected OMCs to river biofilms. We designed different short-term experiments using biofilms exposed to single and combined effects of C_60_ and three different OMC. Specifically, the organic contaminants were selected for their different chemical structure, specific mode-of-action, widespread occurrence in rivers, capacity to bioaccumulate in biofilms and their known toxic effects in freshwater organisms *per se* (**Table 1**) (Kuzmanović, 2015; Huerta et al., 2016). The selected OMC were a pharmaceutical (venlafaxine), a personal care product (triclosan), and a pesticide (diuron), with specific mode-of-actions and different potential toxic effects. We hypothesized that the toxic effects of these OMC on biofilms, when mixed up with fullerenes, would not be homogeneous, but either synergic or antagonistic according to their different chemical structures.

**Table 1:** Chemical and toxic characteristics of the organic micro-contaminants used in this experiment.

Compound		Formula	Molar mass (g/mol)	Log kow^∗^	Log D8^∗^	Major species at pH 8	pKa	EC_50_
Venlafaxine	Psychiatric drug	C_17_H_27_NO_2_	277.40	2.74	1.78	Cation	10.09	EC_50_ 72 h algae = 11,000 μg L^−1^ (Bastos et al., 2017)
Triclosan	Antibacterial	C_12_H_7_O_2_Cl_3_	289.54	4.98	4.50	Anion	7.9	EC_50_ 48 h bacteria = 43.8 μg L^−1^ (Ricart et al., 2010)
Diuron	Herbicide	C_9_H_10_Cl_2_N_2_O	233.09	2.53	2.53	Neutra	13.18	EC_50_ 24 h algae = 13.3 μg L^−1^ (Ricart et al., 2009)

*^∗^Ref: ChemAxon (https://chemicalize.com/ accessed in 12/01/2018).*

## Materials and Methods

### Experimental Design

Three different experiments were performed consecutively using 5-week-old epilithic biofilms. All the experiments consisted in a 72-h exposure of biofilms to the respective contaminants. So forth, we tested the toxicity of biofilm to each contaminant, first separately [fullerenes, venlafaxine (VEN); diuron (DIU); triclosan (TCS)], and second of the respective mixtures of each OMC with fullerenes. Each experiment was performed using 12 glass mesocosms, with 4 different treatments and 3 replicates per treatment. These were (1) a control with biofilms and without OMC or fullerenes (Control); (2) a treatment with biofilms exposed to fullerenes (C_60_); (3) a treatment with biofilms exposed to each OMC (VEN, DIU, or TCS); (4) a treatment with the corresponding mixture of fullerenes and the respective organic contaminant (VENC60, DIUC60, and TCSC60) (Supplementary Figure 1).

The mesocosms were 25 cm in diameter and 15 cm high and hold a central glass cylinder to define an area of 450 cm^2^. Each mesocosm was filled with 4.5 L of rainwater, and water level was kept constant by means of constant water addition (rate 4.5 mL day^−1^) though a peristaltic pump (Ismatec, MCP, 150 W). A glass blade incorporated to a rotor (12 V, 2.2 W, 60 rpm, Philips) constantly moved the water at a constant velocity of 3.4 cm s^−1^ and forced a homogenous flow circulation in the mesocosms. The mesocosms were operated at 20°C temperature and a constant day–night cycle (12-h light/12-h darkness) using LED lamps (Lumina Led 62, 48 W).

The mesocosms were bottom-covered by glass tiles (1.5 cm × 1.5 cm each) colonized with biofilms. The biofilms on the substrata were 5 weeks old and were separately grown in artificial stream channels (2 m long, 10 cm wide, 7.5 cm deep) located besides the mesocosms. The artificial streams received a constant flow of 60 mL s^−1^ of nutrient-poor water and daily cycles of also 12-h light and 12-h darkness. The original biofilm inoculum was obtained from an oligotrophic pristine stream close to the laboratory. Up to 20 glass tiles of colonized biofilms were moved from the artificial streams to each of the mesocosms 12 h before each experiment started.

### Chemicals Preparation

The analytical standards used were diuron (>98%, CAS: 330-54-1, Sigma-Aldrich), venlafaxine hydrochloride (≥98%, CAS: 99300-78-4, Sigma-Aldrich), and triclosan (≥97%, CAS: 3380-34-5, Sigma-Aldrich) (**Table 1**). Stock solutions of 1000 mg L^−1^ for each compound were previously prepared in methanol. The final concentration of methanol in the mesocosms was 0.001%. Nominal concentrations used in the experiments were 10 μg L^−1^ for diuron and triclosan and 50 μg L^−1^ for venlafaxine. The concentrations of each compound were selected following the EC_50_ values reported in literature for diuron and triclosan. The added concentrations of venlafaxine were lower than those predicted by the available EC_50_ values (**Table 1**).

**FIGURE 1:**
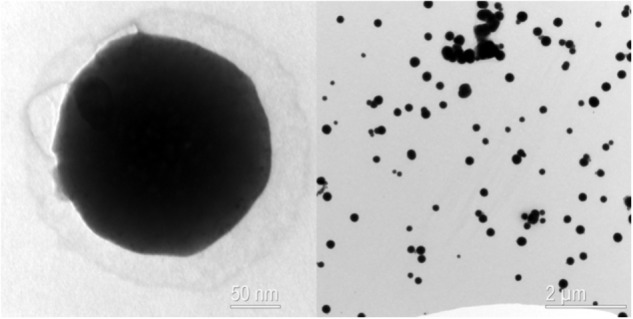
Transmission electron microscopy (TEM) image of fullerene C_60_ from the suspension used in the experiments. The nano-C_60_ aggregates were formed after 2 months stirring in water.

Aqueous stock solution of 100 mg L^−1^ of C_60_ (99.5% Sigma-Aldrich) was prepared using filtered rainwater (0.2-μm pore size) and long-time stirring during 2 months, at constant temperature (20°C) and in absence of organic solvents (Sanchís et al., 2016). Particle size of C_60_ suspension was characterized by transmission electron microscopy (TEM) (Zeiss EM 910). A subsample of stock solution was diluted 10 times with filtered rainwater, sonicated for 1 min, and placed onto 200-mesh grid Formvar membrane. The grid was air-dried and the sample was observed at 60 kV. TEM images (recorded using digital CCD Gatan Orius 200 camera) indicated that the stock solution of C_60_ contained round-shaped aggregates with sizes between 100 and 200 nm, and the particles were clearly dispersed homogeneously after long-time stirring (**Figure 1**).

### Sample Collection

Water samples for nutrient and dissolved organic carbon (DOC) determination were collected at the end of each experiment (after 72 h). Samples were filtered (precombusted 0.45-μm glass microfiber; and 0.2-μm nylon pore size filters, Whatman, respectively) and kept at −20°C until analysis. For quantification of C_60_ and OMC, water samples were collected after 1 h of the initial spiking and at the end of each experiment (time 0 and 72 h). For the analysis of fullerenes, 150 mL of water were filtered by 0.7 μm (glass microfiber filters, Whatman) and then by 0.45 μm (nylon filters, Millipore), the filters were kept −20°C until analysis. For the analysis of the OMC, 10 mL of water were collected in amber glass vials in each mesocosms and kept at −20°C until analysis.

Biofilms were randomly sampled at the end of exposure (72 h) in each of the mesocosms. Triplicate glass tiles were collected from each mesocosm in order to measure organic matter content, extracellular polymeric substances (EPSs), chlorophyll-a (chl-*a*) content, photosynthetic parameters (basal fluorescence and photosynthetic efficiency), extracellular enzyme activities, respiration, and absolute quantification of the expression of 16S and 18S rRNA genes. Chl-*a* content was also measured before any experiment started (time 0 h). Biofilm samples for chl-*a*, EPS, and gene expression were kept at −20°C and −80°C until analysis. The others endpoints were analyzed in fresh during the same day of experiment.

### Water Analysis

Physical variables (pH, oxygen, conductivity, and temperature) were measured using portable hand-held probes (WTW, Weilheim in Oberbayern, Germany) in each mesocosms at the end of each experiment. NO_2_, NO_3_^−^, and NH_4_^+^ were analyzed by ionic chromatography (Dionex, ICS 5000) and PO_4_^3−^ was analyzed spectrophotometrically by the ascorbate-reduced molybdenum blue method. DOC was quantified using a total organic carbon analyzer (Shimadzu TOC-V CSH).

For the analysis of venlafaxine and diuron, 1 mL of water sample was centrifuged (7500 rpm, 10 min, 4°C), then 0.9 mL of supernatant was transferred in a vial, and 0.1 mL of methanol was added. 10 μL of a 1 ng μL^−1^ mixture of isotopically labeled standards solution (VLF-d_6_ and DIU-d_3_) was added before the analysis by liquid chromatography coupled with a hybrid mass spectrometry detector (UPLC-QqLIT) (Gros et al., 2012). For the analysis of triclosan, 1.35 mL of water was mixed with 0.15 mL of methanol. Then, it was centrifuged (7500 rpm, 10 min, 4°C) and 1 mL of supernatant was transferred in a vial. 50 μL of a 1 ng μL^−1^ standard solution of TCS-d_3_ was added before the analysis by UPLC-MS/MS using a methodology adapted from Gorga et al. (2013).

The concentration of fullerenes in water was analyzed using the method thoroughly described in Sanchís et al. (2015). Briefly, fullerenes were extracted from filters by ultrasound-assisted extraction with toluene. The extracts were concentrated to 1.00 mL and analyzed by liquid chromatography coupled to high resolution mass spectrometry. The chromatographic separation was achieved with a Buckyprep column and a non-aqueous mobile phase, composed by toluene–methanol (90–10), in isocratic mode; the ionization was carried out with an atmospheric pressure photoionization source (APPI), working in negative polarity; and the acquisition was performed in full scan mode with a Q Exactive (Thermo Fisher Scientific, San Jose, CA, United States).

### Structural and Functional Biofilm Endpoints

Algal biomass was estimated by extracting chl-*a* with 90% acetone overnight at 4°C in the dark. Biomass was determined spectrophotometrically (Agilent technologies 8453) after filtration of the extract (GF/F, Whatman) by measuring absorbance at 430 and 665 nm (Jeffrey and Humphrey, 1975). Organic matter content was estimated after drying (70°C) during 72 h and then burnt using a muffle furnace (AAF 110, carbolite) for 4 h at 450°C to obtain the ash-free dry weight.

Extracellular polymeric substance was extracted using conditioned cation-exchange resin (Dowex Marathon C, Na^+^ form, strongly acid, Sigma-Aldrich) following the method described in Romaní et al. (2008). The polysaccharide content of biofilm was quantified by the phenol–sulfuric acid assay (Dubois et al., 1956) and measuring the absorbance at 485 nm using a spectrophotometer (Agilent technologies 8453). Glucose standards were also prepared (0–150 μg mL^−1^), and the results were given as glucose equivalents per cm^2^ of biofilm.

*In vivo* chl-*a* fluorescence was used to estimate basal chl-*a* fluorescence (*F*_0_) and PSII photochemical efficiency of the chl-*a* fluorescence (*Y*_eff_) (Kumar et al., 2014). These parameters were measured randomly at five different glass tiles with a portable pulse amplitude modulate fluorometer (Diving PAM, Walz, Germany). Measurements were done for each microcosm at light-adapted state at the same day hour.

Extracellular enzyme activities were determined using artificial fluorescent substrates 4-methylumbelliferone (MUF)-β-D-glucoside, MUF phosphate, and L-leucine-4-7-methylcoumarylamide (AMC), for β-glucosidase (GLU), phosphatase (PHO), and Leu-aminopeptidase (LEU) activities, respectively. One glass tile was incubated at saturating conditions (i.e., 0.3 mM final substrate concentration) for each experiment and mesocosms, in agitation, for 1 h in the dark with filtered mesocosms water (0.2-μm pore size, nylon, Whatman). At the end of the incubation, glycine buffer (1/1, vol/vol) was added to each vial to stop the reaction. The fluorescence of the supernatant was measured into 96-well black microplates at 365/455 nm (excitation/emission) for MUF and 364/465 nm (excitation/emission) for AMC using a fluorometer (Hitachi, F-7000).

We used the MicroResp method for measuring the respiration of biofilm suspensions following the procedure described by Tlili et al. (2011). Briefly, 500 μL of biofilm suspension obtained by scraping two glass tiles with 15 mL of 0.2-μm filtered water from each mesocosms was added to each well (96-well micro-plate). A detection microplate was previously prepared (indicator solution set in a 1% gel of agar, 1:2 ratio) following the manufacturer’s instructions. The two micro-plates (detection plate and biofilm plate) were sealed and incubated in the dark at 20°C in constant agitation (150 rpm) during 24 h. Absorbance was measured at 570 nm (Epoch microplate reader, Bioteck Instruments) immediately before sealing and after the 24 h of incubation. The CO_2_ quantities were calculated using a calibration curve of absorbance values versus CO_2_ quantity measured by gas chromatography. Results were expressed as μg of CO_2_ production rate per gram of ash-free dry weight (AFDW^−1^ h^−1^).

### Molecular Analysis

RNA was extracted after scraping one glass tile per mesocosms using the Power Biofilm RNA isolation Kit (Mo Bio Laboratories, Inc.) according to the manufacturer’s instructions. Aliquots of 50 μL of extracted RNA were purified using a commercial kit TURBO DNA-free TM specifically designed to remove contaminating DNA. Then, SuperScript III for RT-PCR (Invitrogen) was used to synthesize cDNA using 1/2 diluted RNA and 50 ng μL^−1^ random hexamers following the manufacturer’s instructions. RNA and cDNA concentration was measured using Qubit 2.0 fluorometer (Life Technologies).

The genes for 16S ribosomal RNA (rRNA) and 18S rRNA were amplified by real-time PCR (qPCR) using cDNA samples. The primers used for quantification of 16S rRNA were F1048 and R1194 and for 18S rRNA were euk345F and euk499R. All qPCR assays were conducted on an Mx3005P system (Agilent Technologies). All reactions were performed in triplicate and contained a total volume of 30 μL, including 1 μL of cDNA, 1 μL of each specific primer (10 mM), 15 μL of SYBR-Green mix (Brilliant III Ultra-Fast SYBR-Green QPCR Master Mix, Agilent Technologies), and 12 μL of DEPC-treated water. For negative controls, cDNA was replaced by DEPC-treated water. The cycling protocol consisted in initial cycle of 95°C for 3 min, followed by 35 cycles at 95°C for 20 s and 60°C for 60 s for 16S rRNA and 50 cycles at 95°C for 15 s and 60°C for 60 s for 18S rRNA. Standard curves were used to known quantities of cloned target genes, obtained by a series of dilutions following the protocol previous described in Romero et al. (2018). A dissociation curve was constructed to verify the specificity of amplified products obtained during a gradual heating of the PCR products from 60 to 95°C. Results were expressed as number of copy of each gene per ng of cDNA^−1^.

### Data Treatment

Normality of all variables was checked prior to all the analyses by means of Shapiro–Wilk test and Levene’s test for homogeneity of variance, after a log10 transformation. A *t*-test was performed to compare the concentrations of nutrients and OMCs between treatments for each experiment. A generalized linear model (GLM) test was used to detect the individual and main effects (Crain et al., 2008) between C_60_, OMC, and their interactions. The main effects compare the net effect of a stressor (either the C_60_ or a given OMC) in the presence and absence of a second stressor (any contaminant different from the previous, and the control). Individual effects (the response in the presence of a stressor alone vs. the control) were used to calculate the effect size (Crain et al., 2008), from which it derived whether a significant interaction effect occurred against the null model of additively (i.e., the interaction could be resolved as the sum of the individual effects of C_60_ and the respective OMC). When the interactions between C_60_ and each of the OMC pointed to a response significantly different to that additive, the interactive effects were classified as (i) antagonism (A) when the combined effect of C_60_ and the OMCs on a given variable was less than that predicted additively or (ii) synergism (S) when the combined effect of C_60_ and the OMCs on a given variable was more pronounced than that predicted additively. These analyses were conducted in R software version 3.3.0 (R Core Team, 2017) using the *glm* and *t.test* functions.

Principal coordinates analysis (PCoA) based on Bray–Curtis distance matrices was performed including all the functional and structural endpoints. The PCoA is an unconstrained ordination approach aimed to visualize the differences between treatments. Data were used after their previous logarithmic transformation and later fitted to the PCoA plot using Spearman correlations (Blanchet et al., 2008). Finally, an analysis of similarity (ANOSIM) was used to determine statistical differences between each treatment for each experiment separately. These analyses were performed using PRIMER v6 software (PRIMER-E, Ltd., United Kingdom).

## Results

### Water Analysis

The water chemical characteristics remained steady throughout the experiments. Conductivity ranged between 155.4 and 201 μS cm^−1^, pH averaged 8.1 ± 0.2, dissolved oxygen 10.2 ± 1.1 mg L^−1^, and water temperature 19.4 ± 0.1°C (mean ± SD; *n* = 36). The average values for nutrients and DOC concentrations experienced some changes (**Table 2**). While differences between treatments were minor in the case of inorganic nutrients N-NO_2_, N − ${\text{NO}}_{3}^{-}$, N − ${\text{NH}}_{4}^{-}$, and P − ${\text{PO}}_{4}^{3-}$ (except in a few cases, **Table 2**), DOC largely increased at the treatments with venlafaxine (VEN and VENC60) and diuron (DIU and DIUC60) with respect to the Control and the C60 (*t*-test, **Table 2**), and showed a slightly increase in the experiments with triclosan (*t*-test, **Table 2**).

**Table 2:** Nutrients and DOC concentrations for each treatment and experiment (1; venlafaxine, 2; diuron, 3; triclosan).

		DOC mgL^−1^	N-NO_2_ μgL^−1^	N-${\text{NO}}_{3}^{-}$ mgL^−1^	N-${\text{NH}}_{4}^{+}$ μgL^−1^	P-${\text{PO}}_{4}^{3-}$ μgL^−1^
Experiment 1	Control	2.62 ± 0.18	18.55 ± 1.03	1.58 ± 0.07	3.87 ± 0.01	3.75 ± 0.01
	C60	2.38 ± 0.25	16.76 ± 2.30	1.59 ± 0.03	3.87 ± 0.01	4.16 ± 1.38
	VEN	7.41 ± 0.27^∗∗^	23.86 ± 2.15^∗∗^	1.56 ± 0.11	3.76 ± 0.28	2.77 ± 0.01
	VENC60	7.18 ± 0.43^∗∗^	24.46 ± 0.58^∗∗^	1.54 ± 0.09	<LOQ	3.75 ± 0.23
Experiment 2	Control	3.66 ± 0.37	22.41 ± 3.31	1.40 ± 0.09	4.13 ± 0.01	<LOQ
	C60	4.10 ± 0.54	28.77 ± 0.24^∗^	1.32 ± 0.09	3.53 ± 0.01^∗^	3.86 ± 0.90^∗^
	DIU	8.99 ± 1.07^∗∗^	29.07 ± 1.18^∗^	1.50 ± 0.03^∗^	7.28 ± 2.15	5.49 ± 1.97^∗^
	DIUC60	7.76 ± 1.26^∗^	28.40 ± 2.62	1.67 ± 0.01	<LOQ	4.73 ± 0.01
Experiment 3	Control	3.63 ± 0.13	26.47 ± 5.58	1.32 ± 0.07	7.54 ± 2.53	5.60 ± 1.96
	C60	4.52 ± 1.65	27.60 ± 4.95	1.24 ± 0.10	3.94 ± 0.29	3.26 ± 0.01^∗^
	TCS	4.21 ± 0.18^∗^	28.21 ± 6.91	1.21 ± 0.02	<LOQ^∗∗^	3.26 ± 0.12^∗^
	TCSC60	4.23 ± 0.04^∗∗^	25.52 ± 7.66	1.31 ± 0.08	<LOQ^∗∗^	3.75 ± 0.01

*Values are means ± standard deviation (n = 3). The asterisks indicate the significance (t-test, ^∗^p < 0.05; ^∗∗^p < 0.001) for the difference of the treatment values with respect to the control. <Below limit of quantification (LOQ): N-$NH_{4}^{+}$: 0.004 mg L^−1^; P-$PO_{4}^{3-}$: 0.003 mg L^−1^.*

The concentrations of OMC in water decreased after 72 h of exposure (**Table 3**). In the absence of C_60_, the concentrations of venlafaxine significantly decreased by 9%, concentrations of diuron by 13% and concentrations of triclosan by 40% (*t*-test, **Table 3**). In the presence of C_60_, the concentration of diuron decreased by a 4.3 and 12% for venlafaxine (*t*-test, ns), but triclosan concentration decreased significantly by 50% (*t*-test, *p* < 0.01) (**Table 3**). The mean concentration of C_60_ after 72 h was 1.0 ± 0.4 μg L^−1^ (*n* = 18), implying that fullerenes reduced by 64% (mean value) of the initial concentration (**Table 3**). However, the reduction of C_60_ after 72 h was only significant in the TCS treatments (*t*-test, **Table 3**). The occurrence of very low concentrations of C_60_ in the control of TCS and treatment of DIU could be due to air contamination between the mesocosms.

**Table 3:** Fullerenes (C_60_) and organic micro-contaminants (OMCs) concentration, expressed as μg L^−1^, at time 0 h and after 72 h of exposure for each experiment (1; venlafaxine, 2; diuron, 3; triclosan) and treatments.

		C(C60)		C(OMC)	
		*t* = 0 h	*t* = 72 h	*t* = 0 h	*t* = 72 h
Experiment 1	Control	<LOD	<LOD	<LOD	<LOD
	C60	3.08 ± 0.25	1.30 ± 0.07	<LOD	<LOD
	VEN	<LOD	<LOD	56.30 ± 2.13	51.01 ± 1.34^∗^
	VENC60	3.02 ± 0.12	1.07 ± 0.04	49.90 ± 2.83	43.86 ± 1.01
Experiment 2	Control	<LOD	<LOD	<LOD	<LOD
	C60	2.50 ± 0.25	1.20 ± 0.39	<LOD	<LOD
	DIU	<LOD	0.002 ± 0.001	10.29 ± 0.25	8.95 ± 0.15^∗∗^
	DIUC60	2.57 ± 0.04	1.04 ± 0.14	9.72 ± 0.33	9.31 ± 0.96
Experiment 3	Control	<LOD	0.016 ± 0.022	<LOD	<LOD
	C60	1.35 ± 0.03	0.30 ± 0.07^∗^	<LOD	<LOD
	TCS	0.018 ± 0.005	0.017 ± 0.025	8.24 ± 0.95	4.87 ± 0.17
	TCSC60	1.25 ± 0.04	0.37 ± 0.04^∗^	6.80 ± 0.87	3.39 ± 0.30^∗^

*Values are means ± standard deviation (n = 3). The asterisks indicate the significance (t-test, ^∗^p < 0.05; ^∗∗^p < 0.001) for the difference between time 72 h against time 0 h. <Below limit of detection (LOD): venlafaxine (0.009 μg L^−1^); diuron (0.019 μg L^−1^); triclosan (0.012 μg L^−1^); C60 (0.001 μg L^−1^).*

### Structural Endpoints

**FIGURE 2:**
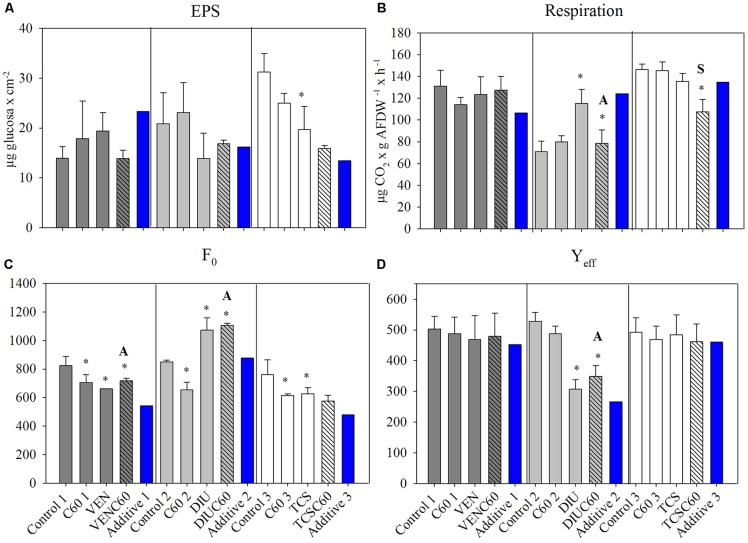
Mean and standard deviation of extracellular polymeric substances (EPSs) **(A)**, respiration **(B)**, basal fluorescence (*F*_0_) **(C)**, and photosynthetic efficiency (*Y*_eff_) **(D)**, measured for the three different experiments after 72 h of exposure (Control, C_60_, each micro-contaminant, and combination of both). Additive effect for each experiment (blue column) is also added, calculated following (Crain et al., 2008). The asterisk indicates a significant *p*-value in GLM analysis. Interactions different than additive are indicated as A (= antagonistic) and S (= synergistic).

No significant differences in chl-*a* content occurred among treatments at time 0 h (data not shown) neither after 72 h of exposition in the three experiments (**Table 4**). EPS content significantly decreased in the TCS treatment with respect to the control (**Figure 2A**). The GLM analysis reported significant individual effects on EPS for TCS, but the interaction between TCS and C_60_ did not differ from the additive response (**Table 4**). *In situ* basal chlorophyll fluorescence (*F*_0_) was responsive to OMC and C60 treatments (**Figure 2C**). *F*_0_ was significantly decreased by C_60_ in all the experiments (**Table 4**). The exposure to OMC decreased the *F*_0_ in VEN and TCS and increased it in the DIU experiment (**Figure 2C**). Significant antagonistic effects in the *F*_0_ occurred when C_60_ interacted with VEN and DIU (**Figure 2C** and **Table 4**).

**Table 4:** Results of the generalized linear model (GLM) for the analyzed endpoints for each experiment and treatment.

Endpoints	Experiment 1 Venlafaxine			Experiment 2 Diuron			Experiment 3 Triclosan		
	C60	VEN	VENC60	C60	DIU	DIUC60	C60	TCS	TCSC60
Chl-*a*	ns	ns	ns	ns	ns	ns	ns	ns	ns
EPS	ns	ns	ns	ns	ns	ns	ns	0.002	ns
*F*_0_	0.015	0.006	0.017	0.002	<0.001	0.005	0.017	0.024	ns
*Y*_ eff_	ns	ns	ns	ns	<0.001	0.047	ns	ns	ns
RESP	ns	ns	ns	ns	<0.001	0.005	ns	ns	0.020
GLU	ns	ns	ns	ns	ns	ns	ns	ns	ns
PHO	ns	ns	ns	ns	0.047	ns	ns	ns	ns
LEU	ns	ns	ns	ns	ns	ns	ns	ns	ns
16S rRNA	ns	ns	ns	ns	0.016	ns	ns	ns	ns
18S rRNA	ns	0.002	ns	ns	ns	ns	ns	ns	ns

*Chl-a, chlorophyll-a; EPSs, extracellular polymeric substances; F_0_, basal fluorescence; Y_eff_, photosynthetic efficiency; RESP, respiration; GLU, β-glucosidase; PHO, phosphatase; LEU, Leu-aminopeptidase activities; ns, no significant effect.*

### Functional Endpoints

The respiration rate (MicroResp technique) significantly increased in the DIU treatment (**Figure 2B** and **Table 4**) while it decreased in the DIUC60 as compared to DIU. Respiration in the DIUC60 was therefore a result of antagonistic interaction (**Figure 2B** and **Table 4**). Respiration decreased in the TCSC60 treatment with respect to the TCS (**Figure 2B**), showing a synergistic response (**Table 4**). The photosynthetic efficiency (*Y*_eff_) was only affected in the biofilms exposed to diuron (DIU and DIUC60 treatment) (**Figure 2D** and **Table 4**) showing an antagonism response (**Figure 2D**). The extracellular enzyme activities (GLU, PHO, and LEU) only showed a significant effect for PHO activity in the diuron treatment (DIU) (**Table 4**).

### Molecular Analysis

**FIGURE 3:**
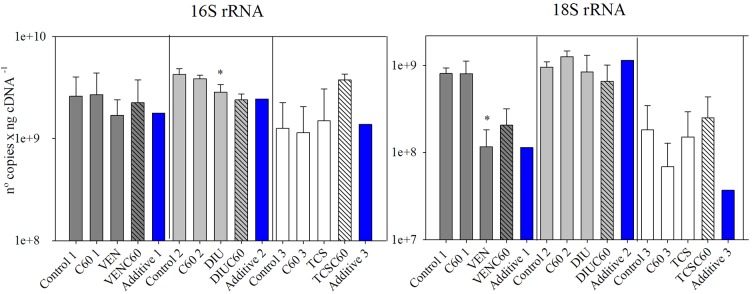
Mean and standard deviation of number of gene copies of 16S rRNA and 18S rRNA measured for the three different experiments after 72 h of exposure (Control, C_60_, each micro-contaminant and combination of both). Additive effect for each experiment (blue column) is also added, calculated following (Crain et al., 2008). The asterisk indicates a significant *p*-value in GLM analysis.

The number of copies of 16S rRNA significantly decreased in the DIU (**Figure 3** and **Table 4**), while the 18S rRNA gene copies experienced a significant decrease in the venlafaxine treatment (VEN). Finally, triclosan did not affect the number of 16S and 18S rRNA gene copies (**Figure 3**).

### Interactive Effects Between C_60_ and Organic Micro-Contaminants

**FIGURE 4:**
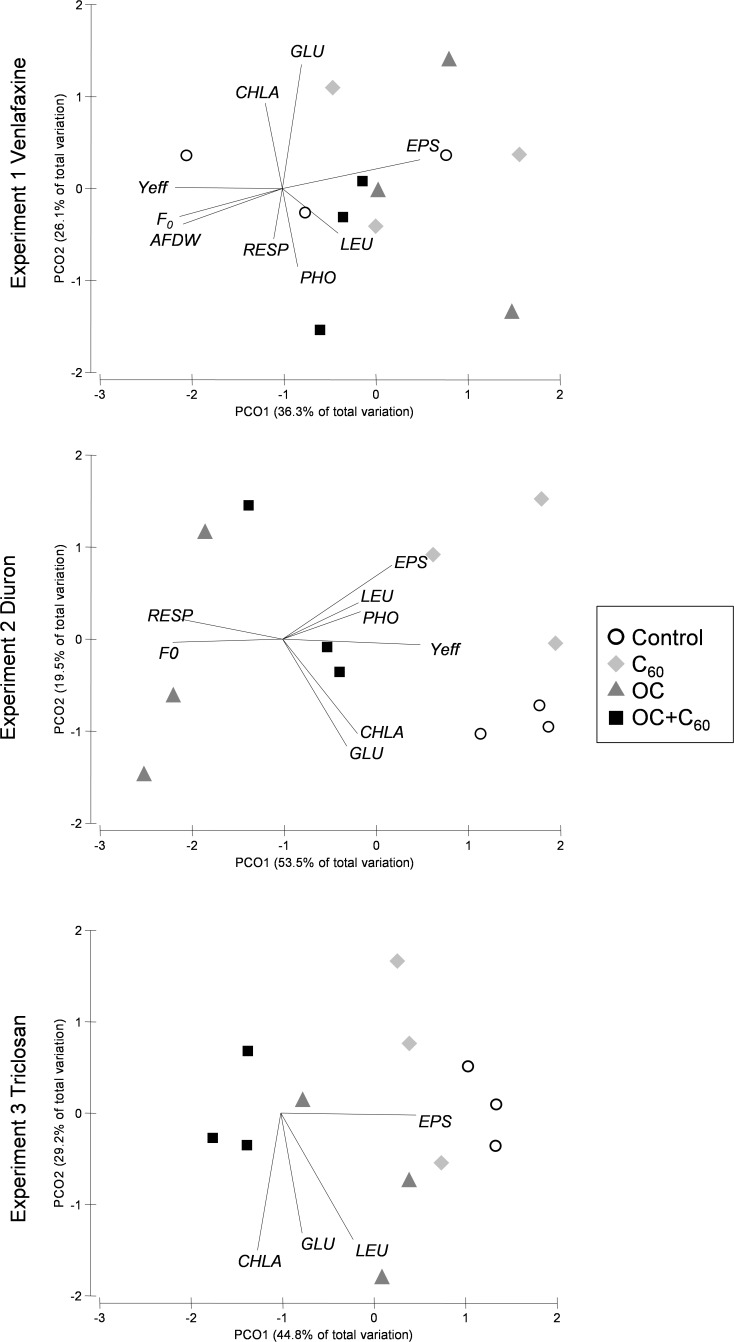
Principal coordinates analysis (PCoA) plot of Bray–Curtis distances between treatments (represent by different colors and symbols) for each experiment including functional and structural endpoints. Significant correlated variables are included in the plot.

The PCoA showed the distinct arrangement of treatments in the diuron and triclosan experiments (ANOSIM, *R* = 0.719, *p* = 0.001 for DIU; *R* = 0.568, *p* = 0.03 for TCS) (**Figure 4**). The DIU samples were more distinctly separated with respect to the control than those of the DIUC60, suggesting that the presence of C_60_ could be associated to a reduction in the toxic effect of diuron. In this analysis, the *Y*_eff_, chl-*a*, and GLU activity had higher loadings in the control samples, while those of respiration and *F*_0_ were higher in the DIU samples. The TCSC60 samples were opposed to those of the control, which had the EPS as the most correlated variable (**Figure 4**). These differences between treatments did not occur in the venlafaxine experiment (ANOSIM, *R* = −0.056, *p* = 0.64) (**Figure 4**).

## Discussion

### Toxic Effects of C_60_ and Organic Micro-Contaminants on River Biofilms

Our results showed that the applied concentrations of C_60_ (ranged between 0.30 and 3 μg L^−1^) did not cause toxic effects to river biofilms, except for the transient response in the biofilm *F*_0_ (basal fluorescence). However, the OMCs produced negative effects on a wide range of structural and functional variables such as EPS, respiration, 16S and 18S gene expression, and extracellular enzyme activities. These different effects of C_60_ and OMCs was not unexpected; toxic C_60_ effects have been described in freshwater microorganisms at concentrations in the range of mg per liter (Lyon et al., 2006; Rodrigues and Elimelech, 2010; Tao et al., 2015; Deryabin et al., 2016; Lawrence et al., 2016), while the concentrations used in our experiment were close to those occurring in the environment (Freixa et al., 2018).

The toxic responses caused by OMC in biofilms changed according to the contaminant and its respective mode of action. The relatively high concentration (∼50 μg L^−1^) of venlafaxine caused significant effects in the *F*_0_ and 18S rRNA gene expression, indicating that algae could be the most concerned (without discarding protozoa or fungi). This chemical has an up to now unknown mode of action on algae, though it acts as a serotonin–norepinephrine reuptake inhibitor and affects the reproduction and metabolism of cladocerans and fish (Henry et al., 2004; Galus et al., 2013; Minguez et al., 2015). On the other hand, diuron inhibits algal photosynthesis by blocking the electron transfer at PSII (Kumar et al., 2014). The negative effects of diuron extend to algal growth and community diversity, as well as to the photosynthetic activity and gene expression (Pesce et al., 2006; McClellan et al., 2008; Ricart et al., 2009; Proia et al., 2011; Moisset et al., 2015). Diuron in our experiments produced a significant reduction of photosynthetic efficiency and a significant increase of basal fluorescence, two previously reported responses in biofilms during long-term experiments (Tlili et al., 2008; Ricart et al., 2009; López-Doval et al., 2010). Diuron also caused a significant reduction of bacterial gene expression (16S rRNA), which probably accounts for the reduction of live bacteria previously observed in biofilm experiments (Ricart et al., 2009). Furthermore, diuron enhanced the CO_2_ production, which could be related to the increase of algal released materials (probably the cause of the large DOC increase in water in this experiment; **Table 2**) and its associated rise in heterotrophic respiration (Pesce et al., 2006). Finally, triclosan caused a significant decrease of EPS content, adding to other structural alterations associated to this bactericide already observed (Lawrence et al., 2009; Morin et al., 2010; Guasch et al., 2016). Such an EPS reduction could be related to a lower bacterial metabolism, which could therefore affect EPS secretion (Lubarsky et al., 2012). Furthermore, triclosan produced a significant decrease in *F*_0_ which probably accounted for the indirect effects on algae (such as diatom mortality or reduction of algal biomass previously described; Lawrence et al., 2009; Morin et al., 2010; Proia et al., 2011, 2013), produced on top of the main effect on the enzymes involved in the fatty acids synthesis in bacterial cells (Heath et al., 1999).

### Interactive Effects of C_60_ With Organic Micro-Contaminants on River Biofilms

Different studies have already shown that organism responses to multiple stressors may account from additive to synergistic or antagonistic responses (Folt et al., 1999; Crain et al., 2008; Côté, et al., 2016). This range of responses has also been observed on aquatic organisms when organic contaminants are mixed with carbon nanoparticles (Baun et al., 2008; Brausch et al., 2010; Schwab et al., 2013; Sanchís et al., 2016). Indeed, in the present study, we observed antagonistic and synergistic responses on the toxic effects of mixture of C_60_ with OMC in river biofilms. In particular, the effects of mixture of C_60_ and venlafaxine could not be differentiated from the separate effects of this contaminant (which were only noticeable on *F*_0_), the mixture of C_60_ and diuron resulted in antagonistic responses in *F*_0_, *Y*_eff_, and respiration, and finally synergistic responses were observed in biofilms exposed to a mixture of C_60_ and triclosan, illustrating how this mixture can increase the toxicity of this contaminant.

The lack of significant interaction in the venlafaxine mixture (except in the *F*_0_) could be related to its higher concentration in relation to the C_60_ (relation 1:44, C_60_:VEN at the end of the experiment). In the other two OMCs, the concentration ratios with C_60_ were more balanced; the relation between C_60_ and OMC were, respectively, of 1:9 in the DIU and 1:7 in the TCS at the end of the experiments. The ratio in the concentrations of carbon nanoparticles and pollutants is of relevance (Hu et al., 2008; Kah et al., 2011; Sanchís et al., 2016), since low concentrations of nanomaterials with respect to the contaminant, as it was the case in the venlafaxine experiment, may produce similar toxicity than the one solely due to the organic contaminant. On the other hand, reducing the concentrations ratio may favor the higher adsorption of organic contaminants and reduce the contaminant bioavailability (Sanchís et al., 2016). The described antagonistic effect of the C_60_ on the toxic effects of diuron on *F*_0_, *Y*_eff_, and heterotrophic respiration (**Table 4**) deserves special attention. The algal materials released by biofilms (due to diuron exposure) could have been absorbed onto the C_60_ materials, therefore reducing their availability for bacterial metabolism. This potential mechanism of antagonism is supported by a slightly lower DOC observed in the mixture condition. Thus, the C_60_ antagonism with diuron could be related to the presence of large C_60_ aggregates competing with diuron molecules through blocking the cell membrane transporters and receptors, and therefore preventing diuron to enter the cells and to exert its toxic effect. Additionally, the diuron adsorbed by C_60_ could be less available to biofilm organisms (Nowack and Bucheli, 2007). Previous studies with other carbon nanoparticles coincide to show that diuron remains adsorbed by carbon nanotubes. This was observed in an experiment with *Chlorella vulgaris* (Schwab et al., 2013) as well as in other with *Pseudokirchneriella subcapitata* in the presence of 1.5 mg L^−1^ black carbon (Knauer et al., 2007).

Finally, the significant reduction of the CO_2_ production in the triclosan and C_60_ mixture suggested that these caused an increase in the triclosan toxicity to biofilms (**Figure 2** and **Table 4**). This synergism could be attributed to the carrier effect of C_60_, which could facilitate the entrance of triclosan inside the biofilm *via* the Trojan horse effect (Limbach et al., 2007; Deng et al., 2017), that is, using the entry provided by nanomaterials into the cells once adsorbed to them. Triclosan molecules loaded to C_60_ could enter inside the biofilm, and subsequently be released inside the organisms thanks to desorption mechanisms (Deng et al., 2017). This might be a likely mechanism, though the adsorption and desorption of OMC and C_60_ are still not well-investigated, and could operate with an OMC and not with another according to their particular physico-chemical characteristics. Similarly, Baun et al. (2008) reported that the presence of C_60_ decreased the EC_50_ (i.e., increased the toxicity) of phenanthrene from 720 to 430 μg L^−1^ for the algae *Pseudokirchneriella subcapitata*. These findings highlight the potential environmental risk of C_60_ because of its capacity to act as a carrier for some organic contaminants.

## Conclusion

Our results show that fullerenes can alter the toxicity of organic contaminants in the river systems. Still, the different responses we observed in the mixtures between contaminants and carbon nanoparticles could be attributed to several mechanisms: (1) differences in the molecular structure of OMC that can influence the sorption equilibrium between C_60_ and contaminants, (2) concentration proportions between OMC and C_60_, and (3) competition of C_60_ contaminants blocking the receptors of the biological cell membranes.

Even though laboratory experiments do not fully capture the ecological complexity of natural aquatic ecosystems, our study contributes to understand the potential effects of fullerenes as modulators of OMCs effects. It is evidenced that C_60_ at environmental concentrations does not only pose a risk for river microorganisms but also that their combination with OMC may produce synergistic and/or antagonistic toxic effects to river biofilms. Our findings suggest that changes in the toxicity of OMC due to the presence of C_60_ in river systems directly affect river biofilms and probably have indirect consequences for river food webs.

## Author Contributions

AF, VA, and SS conceived and designed the study. AF and MG performed the experiments and samplings. AF, MG, JS, and LS analyzed data. AF, VA, JS, LS, SR-M, MF, DB, and SS wrote the manuscript. All authors contributed to the discussion and approved the final version of this manuscript.

## Conflict of Interest Statement

The authors declare that the research was conducted in the absence of any commercial or financial relationships that could be construed as a potential conflict of interest. The reviewer CB and handling Editor declared their shared affiliation.
