# Supplementary material for: Fullerenes Influence the Toxicity of Organic Micro-Contaminants to River Biofilms
Source: Front Microbiol. 2018 Jul 3;9:1426. doi: 10.3389/fmicb.2018.01426 (PMC6037823; doi:10.3389/fmicb.2018.01426)
Supplement: Supplementary file 1 [file Image_1.PDF]

Supplementary Material

FULLERENES INFLUENCE THE TOXICITY OF ORGANIC MICRO CONTAMIMANTS TO RIVER BIOFILMS

Anna Freixa*, Vicenç Acuña , Marina Gutierrez, Josep Sanchís, Lúcia H.M.L.M. Santos, Sara Rodriguez-Mozaz , Marinella Farré , Damià Barceló, Sergi Sabater .

*** Correspondence:** Anna Freixa: afreixa@icra.cat

1.1 Supplementary Figures

A)

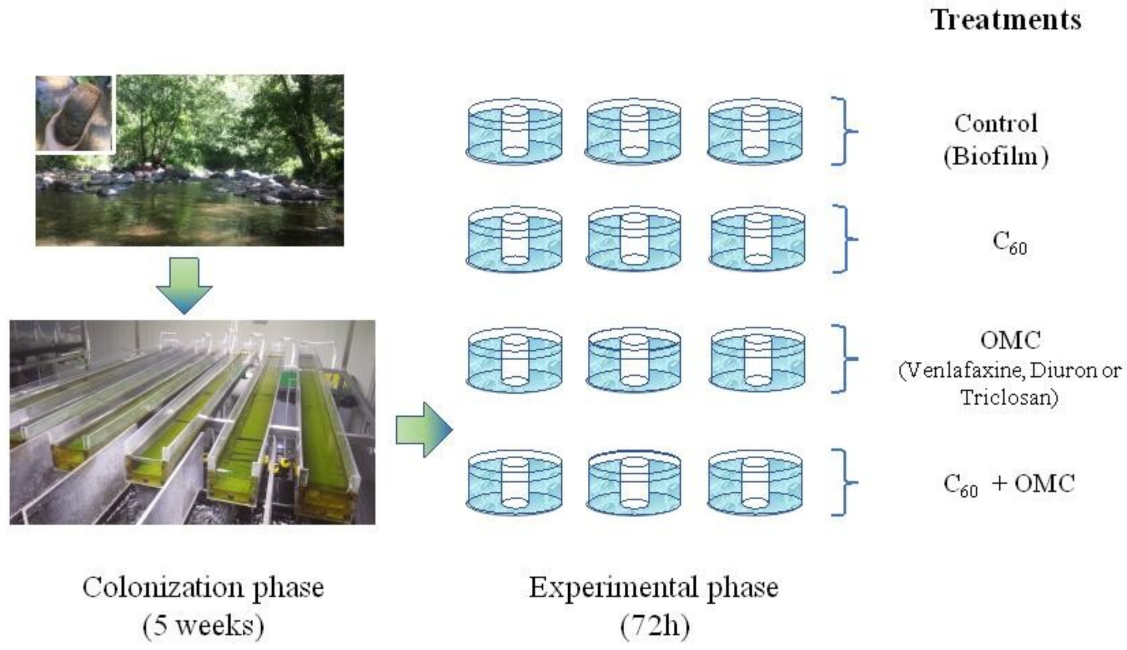

B)

Exp. 1	Control	Exp. 2	Control	Exp. 3	Control
	C_{60}		C_{60}		C_{60}
	VEN		DIU		TCS
	VENC60		DIUC60		TCSC60

Supplementary Figure 1. Scheme of the experimental design of the experiment during colonization and experimental phase showing the 4 different treatments applied. The same experimental procedure was used for each experiment (1: Venlafaxine, 2; Diuron, 3: Triclosan). Panel b showed the different treatments applied for each experiment
